# HIV-1 Replication in Human Immune Cells Is Independent of TAR DNA Binding Protein 43 (TDP-43) Expression

**DOI:** 10.1371/journal.pone.0105478

**Published:** 2014-08-15

**Authors:** Julia Nehls, Herwig Koppensteiner, Ruth Brack-Werner, Thomas Floss, Michael Schindler

**Affiliations:** 1 Institute of Virology, Helmholtz Zentrum Munich, German Research Center for Environmental Health, Neuherberg, Germany; 2 Institute of Developmental Genetics, Helmholtz Zentrum Munich, German Research Center for Environmental Health, Neuherberg, Germany; 3 Institute of Medical Virology and Epidemiology of Viral Diseases, University Clinic Tübingen, Tübingen, Germany; Vita-Salute San Raffaele University School of Medicine, Italy

## Abstract

The TAR DNA binding protein (TDP-43) was originally identified as a host cell factor binding to the HIV-1 LTR and thereby suppressing HIV-1 transcription and gene expression (Ou et al., *J.Virol. 1995, 69(6):3584*). TDP-43 is a global regulator of transcription, can influence RNA metabolism in many different ways and is ubiquitously expressed. Thus, TDP-43 could be a major factor restricting HIV-1 replication at the level of LTR transcription and gene expression. These facts prompted us to revisit the role of TDP-43 for HIV-1 replication. We utilized established HIV-1 cell culture systems as well as primary cell models and performed a comprehensive analysis of TDP-43 function and investigated its putative impact on HIV-1 gene expression. In HIV-1 infected cells TDP-43 was neither degraded nor sequestered from the nucleus. Furthermore, TDP-43 overexpression as well as siRNA mediated knockdown did not affect HIV-1 gene expression and virus production in T cells and macrophages. In summary, our experiments argue against a restricting role of TDP-43 during HIV-1 replication in immune cells.

## Introduction

For effective replication, HIV-1 depends on a variety of host cell factors [1], [2]. On the other hand, mammalian cells also express different factors which may suppress HIV- replication and are collectively termed restriction factors [3], [4]. In recent years, several HIV-1 restriction factors have been identified. These include the cytidine deaminase Apobec3 family members, which cripple HIV-1 genomes by hypermutation, the nucleotide triphosphate hydrolase SamHD1, which inhibits reverse transcription, and Tetherin, suppressing release of virus particles from the cell membrane. These antiviral factors belong to the innate immune response, are mostly interferon triggered and have evolved as a result of the continuous arms race between virus and host [3]. Of note, since restriction factors are part of the ancient cellular antimicrobial armoury they are not virus specific, but act against highly diverse viruses from different families. E.g. Tetherin inhibits release of retroviruses, filoviruses, herpesviruses and members of other enveloped virus families [5]. In addition various host cell factors are known to be capable of suppressing HIV-1 expression from integrated proviruses [6]. These include host transcription factors that bind to the HIV-1 LTR and block viral transcription. While suppressors of HIV-1 expression can limit HIV-1 production in the host, they may also augment the establishment of viral latency and promote virus persistence in the host. Conceivably, a thorough understanding of the mechanisms of viral antagonism by restriction factors could be exploited for the development of novel treatment strategies, and are necessary to finding means to cure HIV infection. Thus, identification and characterization of host cell factors having the potential to inhibit HIV-1 replication is of high relevance.

We here revisited the role of the putative HIV-1 transcription inhibitor TAR DNA binding protein 43 (TDP-43) [7]. In 1995, Ou and coworkers identified TDP-43 from nuclear HeLa cell extracts as a factor capable of binding to HIV-1 LTR TAR DNA probes and recombinant TDP-43 potently suppressed *in vitro* transcription from the HIV-1 LTR [8]. In the meantime, the striking role of TDP-43 in the development of neurodegenerative disorders, e.g. ALS, FTLD, Alzheimer, Parkinson and Huntington disease has become evident. The pathogenesis of these TDP-43 proteinopathies is dependent on a variety of putative TDP-43 functions. Overall, it is now accepted that TDP-43 is a global regulator of transcription and tissue-specific gene expression and TDP-43 has an important role in the development of neurodegenerative disorders called TDP-43 proteinopathies [7], [9], [10] (for a recent review see also [11]). By its RNA recognition motif it is involved in multiple aspects of RNA metabolism, including transcription, splicing, RNA transport, RNA stability and turnover as well as microRNA biogenesis [12]–[14]. Furthermore it appears to be ubiquitously expressed and highly conserved [15]. Taken together, these facts imply that TDP-43 might indeed have the potential to restrict or suppress the gene expression of HIV-1. In addition, deregulation of TDP-43 by HIV-1 could be an important determinant of neurological diseases observed in HIV-1 infected individuals [16].

We investigated a potential role of TDP-43 for HIV-1 replication in infected cell lines and primary cell culture models. While overexpression of TDP-43 had a modest negative impact on HIV-1 LTR transactivation in infected 293T cells, we could not confirm this phenotype in infected T cells. Furthermore, siRNA mediated TDP-43 knockdown did not enhance HIV-1 gene expression or HIV-1 p24 release in infected T cells or primary macrophages. Collectively, our results argue against a role of TDP-43 in repressing HIV-1 infection in human immune cells.

## Results

### HIV-1 infection does not alter the expression and subcellular localization of TDP-43

**Figure 1:**
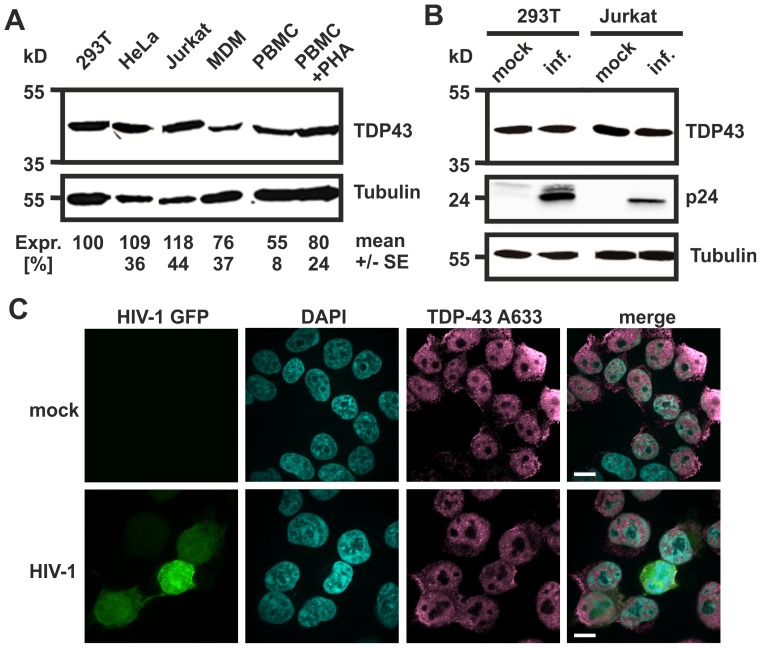
TDP-43 expression and subcellular localization in uninfected and HIV-1 infected cells. (A) The indicated cell lines and primary cells were lysed and analysed by Westernblot for TDP-43 expression. PBMC were either left unstimulated or treated for two days with 10 ng/ml IL-2 and 1 µg/ml PHA. Band intensity of Tubulin and TDP-43 was quantified by densitometric analysis and TDP-43 expression was normalized to Tubulin. Percentages indicate TDP-43 expression relative to 293T cells (set to 100%). Numbers give the mean values and standard error of three independent Westernblots. (B) 293T and Jurkat cells were left untreated or infected with VSV-G pseudotyped HIV-1 IRES-GFP. FACS analysis confirmed infection of >70% of cells. Thereafter, TDP-43, HIV-1 p24 and Tubulin as loading control were detected by WB. (C) Immunofluorescence staining of TDP-43 in HIV-1 GFP infected versus uninfected 293T cells. The scale bar indicates a distance of 10 µm.

TDP-43 is widely expressed [15]. Nevertheless we first aimed to assess TDP-43 protein levels in HIV-1 relevant cell lines and primary cells. Although expression levels varied, TDP-43 was detected in all cell types tested (Fig. 1A). These include 293T cells, which are usually used to generate HIV-1 virus stocks, HeLa cells, Jurkat T cells and primary human monocyte derived macrophages (MDM) as well as resting and PHA stimulated human primary blood mononuclear cells (PBMC). Since all these cells support robust viral replication we hypothesized that HIV-1 might evade the putative restricting TDP-43 activity by inducing its degradation or subcellular sequestration. However, we could neither detect reduction of TDP-43 protein expression in 293T or Jurkat T cells (Fig. 1B) nor changes in subcellular localization of TDP-43 upon infection (Fig.1C).

### Overexpression of TDP-43 modestly suppresses HIV-1 gene expression in 293T cells

**Figure 2:**
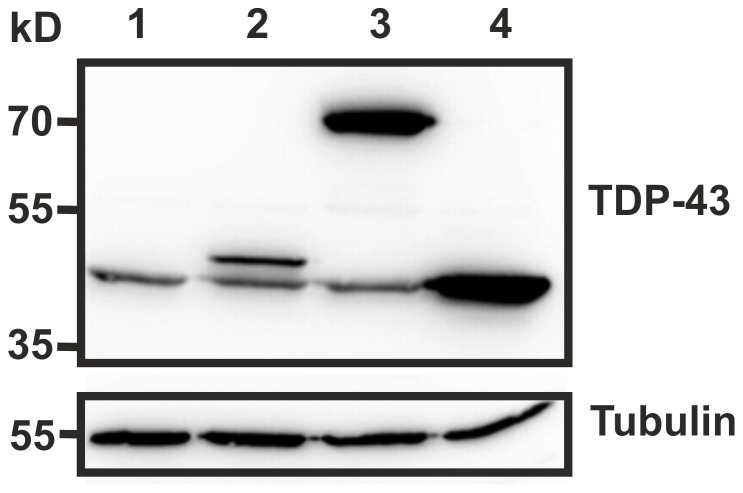
Characterization of TDP-43 expression constructs. Lysates of 293T cells transfected with the indicated TDP-43 expression vectors were analysed for TDP-43 expression by WB. Sample list: 1, mock; 2, TDP-43-V5; 3, TDP-43-CFP; 4, TDP-43-IRES-BFP.

Next, we manipulated cellular TDP-43 expression and simultaneously monitored the consequences for HIV-1 gene expression on a single cell level by flow cytometry. Therefore we utilized CMV promoter driven constructs for expression of TDP-43 either as a fusion protein with V5-tag or CFP, or as an unfused protein together with the blue fluorescent protein (mTagBFP), which is translated from an internal ribosomal entry site (IRES). Functional expression of TDP-43 from the constructs used was assessed by WB (Fig. 2). This analysis further revealed no effect of exogenous TDP-43 expression on the endogenous steady-state protein levels. A possibility we had to explore, since TDP-43 expression might be regulated by a feedback loop [17]. Fusion of TDP-43 with CFP or coexpression of TDP-43 with mTagBFP allows to specifically identify transfected 293T cells by FACS. Then, these cells were infected with *env*-defective VSV-G pseudotyped HIV-1 expressing GFP either together with Nef from an IRES (HIV-IRES-GFP) [18], [19] or inserted into the Gag polyprotein (HIV-iGag-GFP) [20], [21]. In both HIV-1 reporters GFP expression is controlled by the LTR promoter. More specifically, the Nef mRNA is multiply spliced and transcribed early whereas the Gag mRNA is unspliced and transcribed late. See schematic presentations of HIV-IRES-GFP and HIV-iGag-GFP in references [19] and [21]. By this strategy we were able to discriminate putative effects of TDP-43 on early versus late HIV-1 transcription and gene expression.

**Figure 3:**
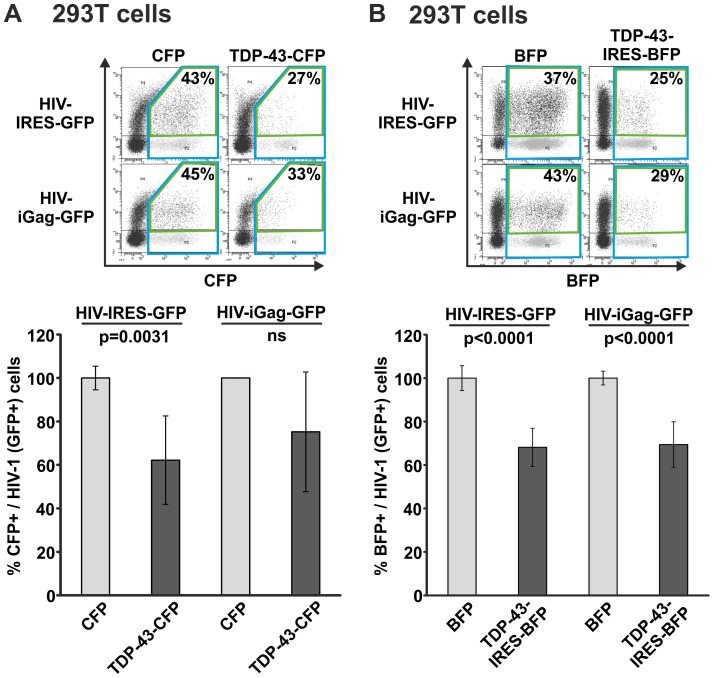
TDP-43 overexpression moderately suppresses HIV-1 gene expression in 293T cells. 293T cells were transfected with (A) TDP-43-CFP expression plasmid or (B) TDP-43-IRES-BFP expression plasmid. One day later cells were exposed to equal amounts of *env*-defective VSV-G pseudotyped HIV-IRES-GFP or HIV-iGag-GFP. 48 hours later levels of GFP were quantified by FACS specifically in the transfected, i.e. CFP/BFP positive population, as marker for viral gene expression. The graph shows mean values and standard deviation from (A) four and (B) five independent experiments. GFP levels in the population transfected with CFP/BFP only were set to 100%. Statistical significant results are indicated by the respective p-value; ns =  not significant.

TDP43-CFP significantly inhibited early gene expression of infected 293T cells by 38%, whereas there was only a trend to inhibition of late gene expression (Fig. 3A). We speculated that the CFP tag might interfere with TDP-43 function. Hence we repeated the experiment but utilized TDP-43 which is translated from a bicistronic mRNA together with mTagBFP. Again, overexpression of TDP-43 resulted in approximately 30% reduction of HIV-1 gene expression, monitored by the levels of GFP for early and late HIV-1 transcripts (Fig. 3B). Altogether, we conclude that overexpressed TDP-43 can modestly decrease HIV-1 gene expression in kidney derived 293T cells.

### TDP-43 does not inhibit HIV-1 gene expression in T cells

**Figure 4:**
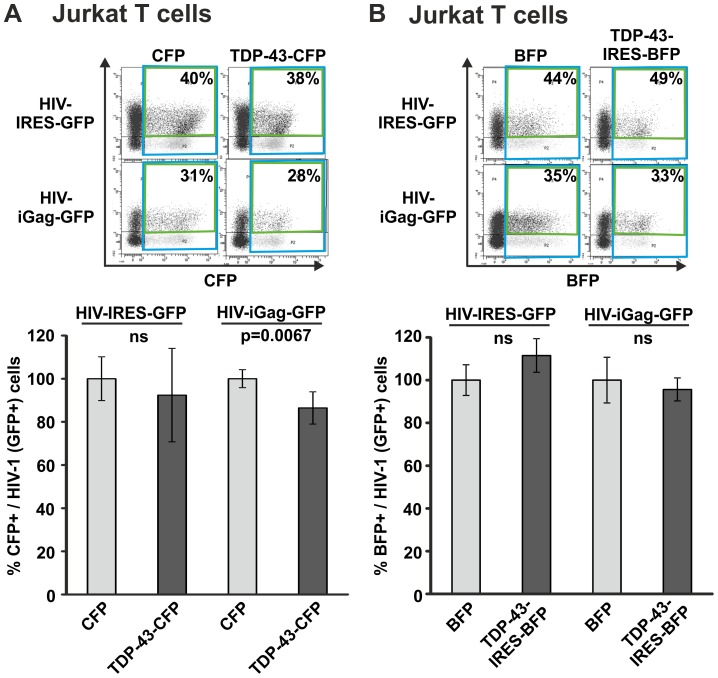
TDP-43 overexpression does not suppress HIV-1 gene expression in Jurkat T cells. Jurkat T cells were microporated with (A) TDP-43-CFP expression plasmid or (B) TDP-43-IRES-BFP expression plasmid. One day later cells were exposed to equal amounts of *env*-defective VSV-G pseudotyped HIV-IRES-GFP or HIV-iGag-GFP. 48 hours later levels of GFP were quantified by FACS specifically in the microporated, i.e. CFP/BFP positive population, as marker for viral gene expression. The graph shows mean values and standard deviation from (A) five and (B) three independent experiments. GFP levels in the population transfected with CFP/BFP only were set to 100%. Statistical significant results are indicated by the respective p-value; ns =  not significant.

In order to assess the effects of TDP-43 in a more HIV-1 relevant cell model we used the CD4+ T cell line Jurkat. Similar to the 293T experiments we microporated Jurkat T cells with the TDP-43 CFP fusion construct or the TDP-43-IRES-mTagBFP vector and the respective control plasmids. Subsequently cells were infected with HIV-IRES-GFP or HIV-iGag-GFP and the number of infected Jurkat T cells in the TDP43 (i.e. CFP/mTagBFP) positive fraction was quantified by assessing the levels of GFP (Fig. 4). In particular, we detected a slight reduction in HIV-iGag-GFP levels in TDP-43 CFP expressing cells (13.5% reduction) that reached statistical significance (Fig. 4A). However in general, overexpression of TDP-43 had no effect on early or late HIV-1 transcription and gene expression in Jurkat CD4+ T cells. Importantly, GFP i.e. HIV-1 expression levels were unchanged or slightly increased in the more relevant experimental setup using untagged TDP-43 (Fig. 4B).

### TDP-43 knock down does not augment HIV-1 gene expression and release

**Figure 5:**
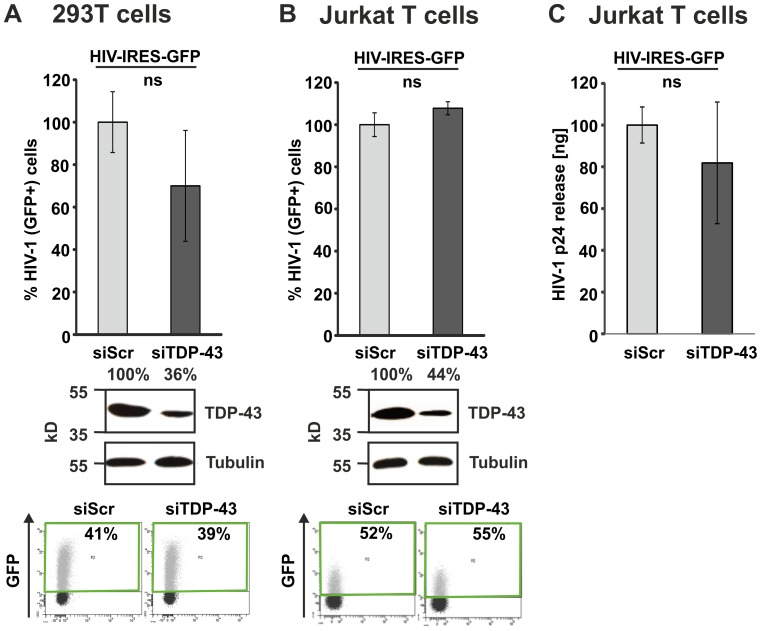
TDP-43 knockdown has no influence on HIV-1 gene expression in 293T and Jurkat T cells. (A) 293T and (B) Jurkat T cells were transfected with a scrambled control siRNA (siScr) or a TDP-43 specific siRNA (siTDP-43). 48 hours post transfection cells were exposed to equal amounts of *env*-defective VSV-G pseudotyped HIV-1 IRES-GFP. Another 48 hours later knock down was confirmed by WB and levels of GFP were quantified by FACS as marker for viral gene expression. The graph shows mean values and standard deviation from (A) three and (B) seven independent experiments. GFP levels in the population transfected with siScr were set to 100%. (C) Quantification of HIV-1 p24 in supernatants from Jurkat T cells treated as described in (B). Levels of p24 in supernatants of HIV-1 IRES-GFP infected Jurkat T cells (n = 3). ns =  not significant.

TDP-43 is tightly regulated and exogenous overexpression might not reflect the consequences of differences in physiological TDP-43 levels on HIV-1 LTR transcription [17], [22]. Hence, we established siRNA mediated TDP-43 knock down. Three to four days post siRNA transfection into 293T or Jurkat T cells we usually observed >50% reduction in TDP-43 levels without toxic effects, as determined by FSC/SSC flow cytometry or MTT test (data not shown). Next, we first suppressed TDP-43 and subsequently infected 293T (Fig. 5A) or Jurkat T cells (Fig. 5B) with HIV-IRES-GFP. In line with the overexpression experiments reduction of endogenous TDP-43 had no detectable effects on the levels of HIV-1 gene expression, as indicated by GFP. To rule out the possibility that TDP-43 restricts HIV-1 production and release at later stages we collected supernatants of Jurkat T cells that were infected with HIV-IRES-GFP and transfected with scrambled or TDP-43 siRNA (Fig. 5C). Quantification of released HIV-1 p24 from the supernatants indicated that TDP-43 did also not suppress HIV-1 production at any stage post gene expression but prior to budding.

**Figure 6:**
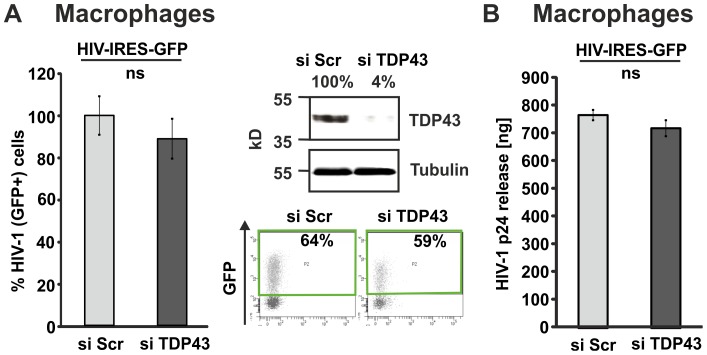
TDP-43 knockdown has no influence on HIV-1 gene expression and p24 release in primary macrophages. (A) Macrophages were preincubated for two hours with Vpx VLPs to increase infection efficiencies. Next, they were exposed to equal amounts of *env*-defective VSV-G pseudotyped HIV-1 IRES-GFP. The next day, macrophages were transfected with a scrambled control siRNA (siScr) or a TDP-43 specific siRNA (siTDP-43). 96 hours post transfection knock down was confirmed by WB and levels of GFP were quantified by FACS as marker for viral gene expression. The graph shows mean values and standard deviation from experiments with macrophages from two donors infected with two independent virus stocks. GFP levels in the population transfected with siScr were set to 100%. Right: (B) Quantification of HIV-1 p24 in supernatants from macrophages treated as described in (A). Levels of p24 in supernatants of HIV-1 IRES-GFP infected macrophages (n = 2). ns =  not significant.

Macrophages are important HIV-1 target cells *in vivo* that potently restrict various steps of HIV-1 infection and production [23]. Hence, we evaluated the effects of TDP-43 in MDM as *ex vivo* primary cell culture model (Fig. 6). In accordance with the results obtained from kidney derived 293T cells or Jurkat CD4+ T cells reduction of endogenous TDP-43 did not alter HIV-1 gene expression (Fig. 6A) or production and release (Fig. 6B) in primary macrophages.

### HIV-1 Tat dependent LTR transactivation is not repressed by TDP-43

**Figure 7:**
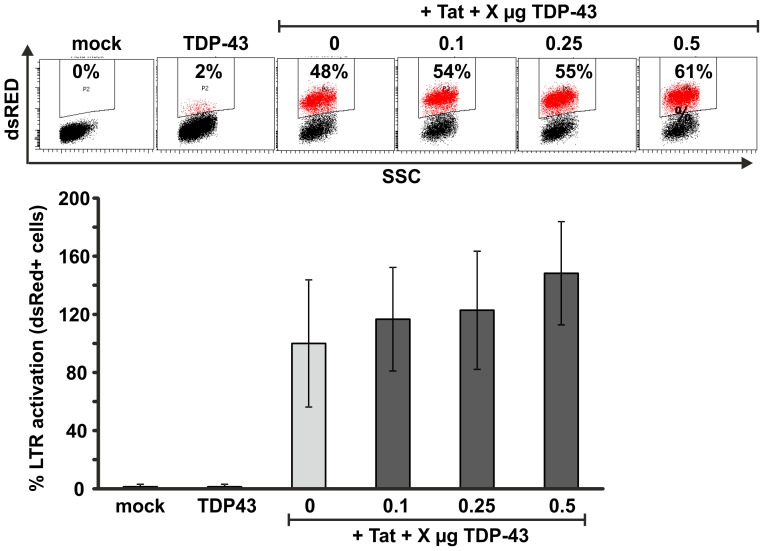
TDP-43 does not suppress HIV-1 Tat mediated LTR transactivation. HeLa-HIV-indi cells expressing dsRed under control of the HIV-1 LTR were transfected with or without HIV-1 Tat and the indicated amounts of TDP-43-V5 expression plasmid. 36–48 hours later cells were trypsinized and dsRed expression was quantified by FACS. The graph depicts mean values and standard deviation of three independent duplicate transfections. The level of dsRed expression induced by Tat without TDP-43 cotransfection was set to 100%.

Viruses evolve countermeasures against cellular restriction factors and we hypothesized that HIV-1 might antagonize TDP-43 by an as yet unknown mechanism. Hence, we designed an infection free experimental setup to detect possible effects of TDP-43 on HIV-1 LTR transcription (Fig. 7). HeLa-HIV-indi cells express a genome integrated HIV-1 LTR that upon transactivation by Tat mediates the expression of dsRed as a fluorescence reporter. Transfection of Tat alone results in approximately 50% of dsRed expressing HeLa-HIV-indi cells as indicator for efficient LTR transactivation. Of note, co-transfection of increasing amounts of TDP-43 did not suppress but slightly enhance the levels of HIV-1 Tat mediated LTR transactivation (Fig. 7). Thus, even in the absence of other viral proteins, TDP-43 is not capable of restricting Tat mediated HIV-1 LTR transcription.

## Discussion

TDP-43 was identified originally in an approach to characterize cellular factors that bind to the HIV-1 TAR DNA region. It was suggested that TDP-43 strongly binds to double-stranded TAR DNA and may repress transcription from the HIV-1 LTR [8]. However, Ou and coworkers used rather artificial approaches to study the function of TDP-43 in the context of HIV-1 gene expression, i.e. adding recombinant TDP-43 to *in vitro* transcription reactions. Thus, the present study focused on elucidating the role of TDP-43 in the HIV-1 replication cycle. We (i) analysed expression levels of TDP-43 in artificial and immortalized HIV-1 relevant host cell lines as well as in primary target cells, (ii) investigated TDP-43 expression levels and subcellular localization in HIV-1 infected cells, (iii) assessed the impact of TDP-43 overexpression and siRNA mediated gene knock down on HIV-1 gene expression and LTR transcription and (iv) clarified whether TDP-43 can suppress HIV-1 Tat mediated LTR transactivation. In summary, our data suggests that TDP-43 does not negatively influence HIV-1 LTR transcription.

In line with previous reports stating that TDP-43 is expressed ubiquitously throughout the human body [15], [24], we demonstrated that TDP-43 is expressed in HIV-1 target cells, such as peripheral blood mononuclear cells (PBMCs), monocyte-derived macrophages and Jurkat T cells. But also rather artificial cell lines used in HIV-1 research like HeLa or 293T cells express TDP-43. Thus, considering TDP-43 as putative HIV-1 restriction factor there would be an urgent need for viral countermeasures.

However, TDP-43 was neither degraded in HIV-1 infected Jurkat T cells or 293T nor translocated from the nucleus. Of note, some HIV-1 restriction factors, i.e. SamHD1, are downregulated upon CD4+ T cell activation. This may be one of the reasons why HIV-1 causes general immune activation and potently replicates in activated but not resting T cells [25], [26]. But again, the fact that TDP-43 levels were unchanged post stimulation of PBMC with PHA argues against a role of TDP-43 in HIV-1 permissiveness of CD4+ T cells.

Gene overexpression and knockdown studies are classical approaches used to analyse the function of a particular protein in living cells. We overexpressed or depleted TDP-43 in 293T, Jurkat T cells and macrophages and the cells were subsequently infected with HIV-1. Infection of TDP-43 overexpressing 293T cells resulted in approximately 30% reduced HIV-1 gene expression levels. In contrast, infection of TDP-43 overexpressing Jurkat T cells did not lead to changes in HIV-1 gene expression levels. Strikingly, these experiments were confirmed multiple times with vectors expressing TDP-43 CFP fusion proteins or untagged TDP-43 expressed along with BFP via an IRES from a bicistronic mRNA. We further used two different HIV-1 reporter constructs expressing GFP either together with Nef [18], [27], representing an early multiple spliced HIV-1 transcript, or together with Gag [20], [21], which represents a late unspliced HIV-1 mRNA transcript. Moreover, TDP-43 knockdown studies revealed that infection of TDP-43 depleted cells does not result in altered HIV-1 gene expression levels at all in 293T, Jurkat T cells and primary HIV-1 infected macrophages. In agreement with these findings, HIV-1 p24 antigen production was not affected upon TDP-43 knockdown in Jurkat T cells and macrophages indicating that also later steps of viral replication were not repressed.

Since the findings of the TDP-43 overexpression and knockdown experiments did not point to an antiviral activity of TDP-43, it was examined if the postulated inhibitory function of TDP-43 on HIV-1 gene expression might be counteracted by a not yet identified HIV-1 mediated mechanism. Due to its high genetic variability, HIV-1 has evolved sophisticated mechanisms to inhibit antiviral host factors and evade the immune response [3], [28]. For example HIV-1 Vif induces the degradation of the antiviral APOBEC3G host protein [29], [30], Vpu counteracts the action of tetherin to support the release of progeny virions [31], [32], and Nef mediates downregulation of MHC-I to evade recognition by cytotoxic CD8+ T cells [33], [34]. To directly analyse the putative connection between TDP-43 and Tat-dependent HIV-1 LTR activation in the absence of any other viral protein, the HeLa-HIV-indi reporter cell line was used expressing dsRED as a consequence of Tat mediated LTR transactivation. However also in this system, keeping in line with all previous experiments, TDP-43 did not repress LTR transcription and hence gene expression.

Taken together, the results of the present study clearly disagree with the previous report [8], which might be explained by the dissimilarity of the experimental approaches that were used to study the function of TDP-43 in HIV-1 infection. Ou and coworkers initially demonstrated that TDP-43 might be capable of repressing HIV-1 gene expression from the LTR using *in vitro* transcription assays. Recombinant TDP-43 and HIV-1 LTR DNA was added to HeLa nuclear extracts, which subsequently led to impaired transcription from the HIV-1 LTR [8]. However, it can be misleading to extrapolate the read out of an assay in a “*test tube*” to the function in its natural cellular context. Besides, Ou et al. showed that released HIV-1 p24 antigen amounts appeared to be reduced following transfection of HeLa cells with TDP-43 and HIV-1 proviral DNA. Indeed, when TDP-43 was overexpressed, we also observed modestly reduced HIV-1 gene expression levels in 293T cells. This did not come as a surprise, since it has been reported that TDP-43 might act in a cell type-specific manner and can be toxic when highly expressed [9], [14], [35]. On the other hand, artificial overexpression of various proteins might have an effect on viral gene expression in such a setting and Ou and colleagues transfected proviral DNA which results in a viral genome residing in the nucleus as an episome. In contrast, infection of cells with HIV-1 leads to genome integration and consequently mimics naturally occurring infections. Thus, transfection of viral DNA is a rather artificial approach that does not reflect transcription from fully integrated HIV-1 genomes.

The experimental approaches used in the present study are more qualified to reveal the physiological function of TDP-43 in the context of HIV-1 infection. The experiments were done using TDP-43 fusion proteins, but also non-fused TDP-43, as protein tags might affect proper protein folding, function and localization. In addition to TDP-43 overexpression, endogenous TDP-43 was knocked down using siRNA. Moreover, TDP-43 overexpressing and TDP-43 depleted cells were HIV-1 infected which imitates the natural steps of viral replication. Most notably, TDP-43 function was examined in Jurkat CD4+ T cells and primary macrophages, both representing important HIV-1 relevant cell models.

Considering the cumulated results of our study, TDP-43 is not relevant for HIV-1 replication and the production of progeny virions from HIV-1 target cells. Thus, TDP-43 is not a restriction factor of HIV-1 gene expression.

## Material and Methods

### Generation of TDP-43 expression plasmids and proviral constructs

The TDP-43 V5 expression plasmid was a kind gift from Dr. Dorothee Dormann (Department of Biochemistry, Ludwig Maximilians University, Munich). The peCFP-C1 TDP-43 fusion construct expressing TDP-43 with an N-terminal CFP tag was essentially generated as previously described [36]. In brief, the TDP-43 open reading frame was amplified with primers introducing a *XhoI* restriction site at the 5′ end a *BamHI* site at the 3′ end together with the linker sequence. Then, the PCR amplified gene was ligated into the peCFP-C1 vector. The pCG TDP-43-IRES-mTagBFP vector was generated by first exchanging the GFP in the pCG-IRES-GFP vector with mTagBFP using the *NheI* and *PpuMI* restriction sites as described beforen [37]. Then, TDP-43 was PCR amplified to introduce flanking *XbaI* and *MluI* sites. Post amplification the PCR product was ligated by standard molecular cloning techniques. All PCR derived inserts were sequenced to confirm their identity. The *env*-defective HIV-1 NL4-3 IRES-GFP and HIV-1 NL4-3 iGAG-GFP proviral constructs have been described before [18]–[21], [27].

### Cell culture, generation of HIV-1 stocks and infection

293T cells were cultured in in DMEM with 10% FCS and standard supplements. CD4+ Jurkat Tag cells [38] were a kind gift from Prof. Oliver Fackler (Heidelberg) and cultured in RPMI plus standard supplements. PBMC and macrophages were generated from Buffy coat by ficoll density centrifugation and cultured as described [21], [27]. For the generation of virus stocks, 293T cells were transfected by the Calcium phosphate method as before [27]. In brief, 350.000 cells were seeded per six well and transfected with 4.8 µg of proviral DNA. Since we wanted to have single round infections we used *env*-defective versions of our GFP HIV-1 reporter constructs. Thus, 0.2 µg of VSV-G expression plasmid was cotransfected to each well. Six hours later media was changed and additional 24–30 hours later the supernatants were harvested, cleared by centrifugation and directly used for HIV-1 infection experiments. Same volumes (500 µl) of HIV-1 containing supernatants were used to infect 175.000 293T cells in a twelve-well or 1*10∧6 Jurkat cells. This usually resulted in HIV-1 infection rates ranging from 40–60% of GFP+ cells as estimated by FACS analysis. For macrophage infection we treated the cells two hours before infection with Vpx containing VLPs (kind gift of Prof. Thomas Gramberg, Erlangen) to enhance HIV-1 infection rate [39].

### Flow cytometry

FACS analyses were generally done on a FACS CantoII (Becton Dickinson). In brief, cells were resuspended and washed with PBS. HIV-1 infected cells were fixed for 1 h with 2% PFA. Intact cells were identified based of FSC/SSC. CFP and mTagBFP were excited with the 405 nm laser and detected with a 450/50 nm filter whereas GFP was excited at 488 nm and detected by a 530/30 filter. A minimum of 10.000 intact cells were analysed per sample.

### TDP-43 overexpression and siRNA knock down experiments

To analyse the effects of TDP-43 overexpression for HIV-1 gene expression 175.000 293T cells were seeded in 12 well plates and CaCl transfected with 3 µg TDP-43 expression plasmid or the CFP/mTagBFP only vector control. One day later cells were infected with same amounts of HIV-1 GFP. Additional 48 hours later cells were harvested and the percentage of GFP+ cells was determined in the CFP/mTagBFP+ population. The experiment in Jurkat cells was essentially done identically, besides the fact that the TDP43 and control plasmids were delivered by microporation using the NEON device (Life technology). Electroporation parameters were: 3 pulses at 1350 V and 10 msec. We used scrambled control siRNA and TDP-43 specific siRNA with the lead strand sequence 5′-ACCAAAUCCACCCUGAUUCCCCC-3′ from riBOXX. siRNA transfection was done with Interferin (Polyplus) by the standard protocol provided from the manufacturer. For 293T we used 10 nM, Jurkat cells 3 nM and for macrophages 200 nM siRNA. Two days post infection, cells were infected with HIV-1 GFP reporters and analysed by FACS 48 hours later. Knock down was found to be non-toxic by MTT test and most efficient four days post transfection.

### Westernblot and ELISA

Cultivated cells were lysed using 50 µl RIPA buffer and denatured at 95°C for 10 min using 5x SDS sample buffer. The samples were loaded to 10% or 12% polyacrylamide gels and SDS-PAGE was performed at 160 V for one hour. The proteins were then transferred to nitrocellulose membranes using the wet transfer technique at 330 mA for one hour. Following protein transfer, membranes were blocked for approximately one hour with 5% milk powder in TBST. Primary antibodies were diluted in 5% milk and left overnight at 4°C (anti-TDP-43: 1∶1000 (Abcam); anti-p24: 1∶3000 (Abcam); anti-alpha-tubulin: 1∶10000 (Sigma)). The next day, secondary antibodies were prepared and membranes were incubated for 3 hours with antibody solutions (anti-mouse: 1∶10000 (Sigma)). Protein bands were finally visualized using the SuperSignal West Pico Chemiluminescent substrate according to the manufacturer's protocol. Determination of HIV-1 p24 was done with the p24 ELISA obtained from the NCI (Frederick, USA) or the HIV-1 p24 antigen capture assay (Advanced BioScience Laboratories) and according to the provided manuals.

### Immunofluorescence staining

100.000 293T cells were seeded to 12 well plates on poly-L-lysine coated cover slips. Two days later, cells were fixed for 20 min at 4°C with 2% PFA. For permeabilization cells were incubated in 0.5% Triton in PBS for 15 min at RT. Subsequently, unspecific antibody binding sites were blocked for 45 min at room temperature using 5% goat serum. Anti-TDP-43 primary antibodies (Abcam) were diluted 1∶100 in 1% BSA and cells were incubated in a humidity chamber for 1 hour at room temperature. Next, anti-mouse-Alexa-633 secondary antibodies (Invitrogen) were diluted 1∶500 in 1% BSA and cells were incubated for 20 min at room temperature. Finally, cell nuclei were stained for 10 min at room temperature using DAPI diluted 1∶1000 in PBS. Cover slips were mounted onto microscope slides using Mowiol overnight and subsequently imaged by SD confocal microscopy

### Confocal microscopy

Spinning disc confocal microscopy was done with an inverted Nikon TiE microscope equipped with the UltraViewVox System (Perkin Elmer). Microscopic images were generated at 100x magnification. GFP was excited with a 488 nm laser, Alexa633 with a 633 nm laser and DAPI with a 405 nm laser.

### Tat mediated LTR transactivation in the presence of TDP-43

HeLa-HIV–indi cells, stably expressing dsRED-quick under the control of the HIV-1 LTR promoter, were generated by transfection of the pIndi plasmid [40] into HeLa cells with Fugene6 (Promega) according to the protocol provided by the manufacturer. Cells were selected with 0.5 mg/ml Geneticin and single cell clones were generated from the expanded cells. 1*10∧5 HeLa-HIV–indi cells were seeded in 24 well plates one day before transfection. For transfection, a total of 1 µg plasmid DNA was transfected with Metafectene (Biontex) as recommended by the manufacturer. 48 hours later cells were trypsinized, washed with PBS and dsRED expression was quantified by flow cytometry.

### Statistical analysis and software

All statistical analyses were done using GraphPad Prism 5.0. To assess significance values we used the two-tailed unpaired Students T test. Westernblot band intensity was quantified with Image J.
